# Review of: Coal, Cages, Crisis: The Rise of the Prison Economy in Central Appalachia

**DOI:** 10.13023/jah.0503.08

**Published:** 2023-12-01

**Authors:** Ted Olson

**Affiliations:** East Tennessee State University

**Keywords:** Appalachia, book review, incarceration and health, justice-involved populations, prison economy

## Abstract

**Ted Olson**, PhD, is a professor of both Appalachian Studies and Bluegrass, Old-Time and Roots Music Studies at East Tennessee State University. In this piece, he reviews Professor Judah Schept’s *Coal, Cages, Crisis: The Rise of the Prison Economy in Central Appalachia* and discusses the impacts of incarceration on the health of Appalachia and on its residents more broadly.

## CITATION

Schept J. *Coal, cages, crisis: The rise of the prison economy in Central Appalachia*. New York: New York University Press, 2022. ISBN-13: 978-1479858972; Cost: $32.00 paperback, $26.59 Kindle edition, $99.00 Hardcover.

## ABOUT THE REVIEWER

**Ted Olson**, PhD, is a professor of both Appalachian Studies and Bluegrass, Old-Time and Roots Music Studies at East Tennessee State University. He has received nine Grammy nominations for his research into Appalachian music history, and in 2021, he received the highest honor of the East Tennessee Historical Society, the Ramsey Award for Lifetime Achievement. Olson has been a Fulbright Senior Scholar (2008); President of the Tennessee Folklore Society (2003–2005); Co-chair of the curatorial committee for the Smithsonian Folklife Festival’s “Appalachia: Heritage and Harmony” exhibition (2003); and more recently, Co-producer and Co-host of *Sepia Tones*, a podcast series on Black Appalachian music for the Great Smoky Mountains Association.

## ABOUT THE AUTHORS

Judah Schept, PhD, is an associate professor at Eastern Kentucky University’s School of Justice Studies. His research—shaped by Schept’s time at Indiana University (PhD, Criminal Justice) and Vassar College (BA, Sociology) in addition to life in Kentucky—focuses on the prison industrial complex, particularly in Central Appalachia. Schept’s work can be seen across journals such as *Radical Criminology* and *Punishment and Society* and has been featured in talks across the U.S. and in places as far flung as Oxford University, where he delivered a lecture earlier this year.

## THE REVIEW

While posing larger questions about the future of Central Appalachia, *Coal, Cages, Crisis1* focuses on the recent surge of prison construction within the region as an economic replacement for a declining coal industry. The book has its origins in a 2012 incident. As author Judah Schept (a professor in the School of Justice Studies at Eastern Kentucky University) recounts, he and photographer Jill Frank attempted to document—from the outside—a recently constructed state prison in rural Eastern Kentucky (the Little Sandy Correctional Complex in Elliott County). Motivated by both professional and personal concern for the proliferation of prison construction in Central Appalachia, Schept and Frank were attempting to assess the impacts of that prison on the “economically distressed” county (according to the Appalachian Regional Commission) in which it was built. A roving correctional officer saw and accosted the two journalists, threatening to confiscate their equipment and their film; the officer ultimately demanded to see their driver’s licenses. The officially sanctioned harassment may have compelled them to leave the site in order to avoid arrest, but that action had the effect of deepening the journalists’ commitment to investigating Central Appalachia’s “prison economy.” The resulting research—on-the-ground investigative reportage, sociological analysis, and photography—would result, ten years later, in this hard-hitting book.

Acts of aggression by elites toward working-class locals as well as interested outsiders have been all too common in those sections of Appalachia where societally privileged people are allowed—even encouraged—to take matters of social negotiation into their own hands. This sad fact was dramatically represented in *Stranger with a Camera*,^2^ a 2000 film that investigated the circumstances surrounding the 1967 killing of a Canadian filmmaker by an Eastern Kentucky landlord. The filmmaker was interviewing a coal miner—with that miner’s permission—in a rental cabin owned by a man not present at the time of the filmmaker’s arrival. Prompted by neighbors who distorted the nature of the documentary activity, the landlord grabbed a gun; later, he received an abnormally light sentence for the act. Made by an Eastern Kentuckian (Elizabeth Barrett) whose stated goal was to objectively explore a historic incident that had haunted a community, *Stranger with a Camera* revealed deeply entrenched inequities hard baked into Central Appalachia’s socioeconomic power structure. Working in the same Appalachian subregion half a century later and conducting their documentary work within the same empathic media tradition, Schept and Frank—the latter’s black-and-white photographs, scattered strategically across *Coal, Cages, Crisis*, contribute a striking visual perspective on this book’s thesis—shine an unflinching light on a contemporary manifestation of a historic syndrome resulting from the denial of democratic protocols and from the practice of one sector of society tolerating, even promoting and protecting, disparities in access to justice, power, natural resources, and health care.

To examine the emergence of the prison economy in Central Appalachia, *Coal, Cages, Crisis* explores several case studies. In one particularly trenchant chapter, Schept traces the history of the Brushy Mountain State Penitentiary, built in a remote section of East Tennessee’s Cumberland Plateau (Morgan County) at the end of the 19^th^ century. In 1891, striking miners rebelled against nearby coal companies. The book quotes historian Perry Cotham: “The Coal Creek rebellion . . . was nothing less than a working class uprising against upper class management and politicians.” The Brushy Mountain State Penitentiary was built shortly afterward to bring into the area a population of prisoners—most of them Black—who were required as part of their prison sentence to mine coal for the State of Tennessee. Politicians argued for this arrangement as a way to counter the unionization efforts of non-incarcerated miners, with officials believing the presence of a competing workforce might scare striking miners back to their coal jobs. The Coal Creek uprisings, however, resulted in discrimination along racial lines, as striking miners stormed the coalfields in that part of Tennessee and forced Black convicts onto trains for transporting to Knoxville. Schept later discusses the 21^st^ century conversion of this prison into the Brushy Mountain Development site, which officially repurposed the prison to serve as a touristic destination combining guided prison tours, craft liquor sales, and other recreational activities.

Elsewhere, Schept analyzes the positions of prison advocates, who have justified prison-building as a mechanism for creating jobs and for encouraging economic development. He examines how one prison project in Letcher County, Kentucky, was officially proposed despite local resistance, with the project subsequently postponed because of national politics (conflicting budgetary priorities of the Republican Party leadership during President Donald Trump’s term in office), leaving the funding ($444 million) “suspended in legal limbo, appropriated specifically for the project within the Department of Justice budget and thus not able to be spent elsewhere” (223).

While Schept’s political analysis and historical perspective are helpful toward advancing public understanding of the contexts for the ongoing prison economy movement in Central Appalachia, *Coal, Cages, Crisis* offers a more universal revelation: that a community’s healthfulness is directly dependent upon environmental balance, economic sustainability, and social justice.

As this book reveals, those humanistic values are increasingly imprisoned in Central Appalachia within an economic system that in recent years has extracted the region’s natural resources through the most environmentally destructive method imaginable (“mountaintop removal coal mining”), with dire consequences to human health and safety. With decreasing demand for those resources, this economic system has resorted to imposing the prison economy upon a population that has prided itself on its sociopolitical and economic independence. Because its complex narrative is grounded in detail and is presented by means of a trustworthy, informed scholarly approach, *Coal, Cages, Crisis* is an essential exposé of this unfolding predicament in Central Appalachia. Indeed, the book has applicability to other places in which economies are determined by elites with little or no input from the majority of the people.
